# Nitrative and oxidative DNA damage in infection-related carcinogenesis in relation to cancer stem cells

**DOI:** 10.1186/s41021-016-0055-7

**Published:** 2017-01-01

**Authors:** Shosuke Kawanishi, Shiho Ohnishi, Ning Ma, Yusuke Hiraku, Shinji Oikawa, Mariko Murata

**Affiliations:** 1 Faculty of Pharmaceutical Sciences, Suzuka University of Medical Science, Suzuka, Mie 513-8670 Japan; 2 Faculty of Nursing, Suzuka University of Medical Science, Suzuka, Mie 513-8670 Japan; 3 Department of Environmental and Molecular Medicine, Mie University Graduate School of Medicine, Tsu, Mie 514-8507 Japan

**Keywords:** 8-OHdG, 8-oxodG, 8-nitroguanine, Oxidative stress, Inflammation

## Abstract

Infection and chronic inflammation have been recognized as important factors for carcinogenesis. Under inflammatory conditions, reactive oxygen species (ROS) and reactive nitrogen species (RNS) are generated from inflammatory and epithelial cells, and result in the formation of oxidative and nitrative DNA lesions, such as 8-oxo-7,8-dihydro-2’-deoxyguanosine (8-oxodG) and 8-nitroguanine. The DNA damage can cause mutations and has been implicated in inflammation-mediated carcinogenesis. It has been estimated that various infectious agents are carcinogenic to humans (IARC group 1), including bacterium *Helicobacter pylori* (*H. pylori*), viruses [hepatitis B virus (HBV), hepatitis C virus (HCV), human papillomavirus (HPV) and Epstein-Barr virus (EBV)] and parasites [*Schistosoma haematobium* (SH) and *Opisthorchis viverrini* (OV)]. *H. pylori*, HBV/HCV, HPV, EBV, SH and OV are important risk factors for gastric cancer, hepatocellular carcinoma, nasopharyngeal carcinoma, bladder cancer, and cholangiocarcinoma, respectively. We demonstrated that 8-nitroguanine was strongly formed via inducible nitric oxide synthase (iNOS) expression at these cancer sites of patients. Moreover, 8-nitroguanine was formed in Oct3/4-positive stem cells in SH-associated bladder cancer tissues, and in Oct3/4- and CD133-positive stem cells in OV-associated cholangiocarcinoma tissues. Therefore, it is considered that nitrative and oxidative DNA damage in stem cells may play a key role in infection-related carcinogenesis via chronic inflammation.

## Background

Infection and chronic inflammation have been recognized as important risk factors for carcinogenesis and malignancies [1–3]. The International Agency for Research on Cancer (IARC) has estimated that approximately 18 % of cancer cases worldwide are attributable to infectious diseases caused by bacteria, viruses, and parasites [4]. Human cancer caused by infectious agents is shown in Table 1. The following ten infectious agents have been classified as group 1 carcinogens (carcinogenic to humans) by IARC: bacterium *Helicobacter pylori* (*H. pylori*), viruses [hepatitis B virus (HBV), hepatitis C virus (HCV), human papillomavirus (HPV), Epstein-Barr virus (EBV), human T-cell lymphotropic virus type 1 (HTLV-1) and human immunodeficiency virus-1 (HIV-1)] and parasites [*Schistosoma haematobium* (SH), *Opisthorchis viverrini* (OV) and *Clonorchis sinensis* (CS)] [4, 5]. Inflammation can be induced not only by chronic infection, but also by many other physical, chemical and immunological factors [6]. It has been estimated that chronic inflammation accounts for approximately 25 % of human cancers [6]. Cancer risk is heavily influenced by environmental factors such as infections. HPV, HBV/HCV and *H. pylori* may be responsible for about 90 % of cervical cancer cases, 80 % of hepatocellular carcinoma cases and 65–80 % of gastric cancer cases, respectively [7].

**Table 1:** Human cancer caused by infectious agents worldwide and possible markers

Infectious agents	Cancer site	Number of cancer cases	Cancer cases world wide (%)	Detection of 8-nitroguanine [Refs.]	Possible markers for CSC [20]
Bacteria					
*H. pylori*	Stomach	490,000	5.4	Patients [31, 76]	SALL4, KLF5, LgR5
Viruses					
HPV	Cervix and other sites	550,000	6.1	Patients [52]	CK17 CD44 (HPV16) Oct3/4 (HPV16)
HBV, HCV	Liver	390,000	4.3	Patients with HCV [39] Mice with HBV [Fig. 5B, unpublished data]	CK19 Nanog, CD133
EBV	Lymphoma Nasopharyngeal carcinoma	99,000	1.1	Patients [57]	LMP2A LMP1, Bmi-1
HHV-8	Kaposi sarcoma	54,000	0.6		
HTLV-1	Leukemia	9,000	0.1		
Parasites					
SH	Bladder	2,700	0.1	Patients [21, 22]	Oct3/4 (patients with SH) [21] CD44v6 (patients without SH) [22]
Liver flukes	Cholangiocarcinoma	800			
OV				Hamsters [68–70, 75] Patients [71, 74]	CD133, Oct3/4 [74]
CS					
	Total infection- related cancers	1,600,000	17.7		
	Total cancers in 1995	9,000,000	100		

Abbreviations: *CSC* cancer stem cell, *Refs.* references

### DNA damage in inflammation-related carcinogenesis

Under inflammatory conditions, reactive oxygen species (ROS) and reactive nitrogen species (RNS) are generated from inflammatory and epithelial cells. ROS and RNS are capable of causing damage to various cellular constituents, such as nucleic acids, proteins and lipids. ROS are generated from multiple sources, including inflammatory cells, carcinogenic chemicals and their metabolites, and the electron transport chain in mitochondria [2, 3]. ROS can induce the formation of oxidative DNA lesion products, including 8-oxo-7,8-dihydro-2’-deoxyguanosine (8-oxodG), which is considered to be mutagenic [8].

Nitric oxide (NO) is synthesized by NO synthases. There are three isoforms, neuronal NO synthase (nNOS, also known as NOS1), inducible NO synthase (iNOS or NOS2) and endothelial NO synthase (eNOS or NOS3) [9, 10]. iNOS is activated to drastically generate NO in inflammatory and epithelial cells under inflammatory conditions, while eNOS and nNOS are constitutively expressed and produce relatively small amounts of NO. iNOS can be also up-regulated by transcription factors such as NF-kB, HIF-1α, STAT, tumor necrosis factor-α (TNF-α). NF-kB plays a central role in inflammation through its ability to induce transcription of proinflammatory genes, including iNOS, and functions as a tumor promoter in inflammation-associated cancer [11].

**Fig. 1:**
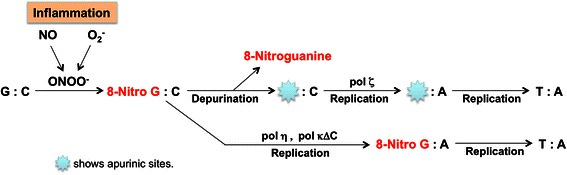
Proposed mechanism of mutation mediated by 8-nitroguanine formation

Figure 1 shows 8-nitroguanine formation under inflammatory conditions and resulting mutation. NO reacts with superoxide (O_2_^−^) to form peroxynitrite (ONOO^−^), a highly reactive species causing 8-oxodG and 8-nitroguanine [12]. The reaction of guanine with ONOO^−^ forms 8-nitroguanine as the major compound, while adenine nitration is minor compared to its C8-oxidation [13]. The glycosidic bond between 8-nitroguanine and deoxyribose is chemically unstable, and this DNA lesion can be spontaneously released, resulting in the formation of an apurinic site [14]. The apurinic site can form a pair with adenine during DNA synthesis, leading to G:C to T:A transversions [15]. In addition, translesion DNA polymerases were discovered and their role in the mutagenesis has been investigated [16]. Cells deficient in Rev1 and Rev3, subunits of DNA polymerase ζ, were hypersensitive to nitrative stress, and translesion DNA synthesis past apurinic sites mediated by this polymerase might contribute to extensive point mutations [17]. It has been reported that adenine is preferentially incorporated opposite 8-nitroguanine during DNA synthesis catalyzed by polymerase η and a truncated form of polymerase kin a cell-free system, suggesting that G:C to T:A transversions can occur [18].

8-Nitroguanine is considered to be not only a marker of inflammation, but also a potential mutagenic DNA lesion involved in carcinogenesis [19]. We have investigated the formation of 8-nitroguanine and 8-oxodG in various clinical specimens and animal models in relation to inflammation-related carcinogenesis, as summarized in Table 1. When specimens or cultured cells were pretreated with RNase, 8-nitroguanine was more clearly observed in the nuclei of cells by immunostaining. It suggests that 8-nitroguanine is formed mainly in genomic DNA. It is noteworthy that nitrative and oxidative DNA lesions were specifically induced at cancer sites under chronic infection and various inflammatory conditions, as reviewed previously [2, 3, 20]. We demonstrated that 8-nitroguanine was strongly formed via iNOS expression at related cancer sites of *H. pylori*, HBV, HCV, HPV, EBV and SH, OV [2, 3, 21, 22]. The IARC classification of CS has been recently updated from 2A to 1, so we have not yet collected enough data for 8-nitroguanine.

Nitrative and oxidative stresses cause DNA damage, contributing to the accumulation of mutations in tissues throughout the carcinogenic process. Particularly, 8-nitroguanine formation may participate in inflammation-related carcinogenesis as a common mechanism. Therefore, 8-nitroguanine could be used as a potential biomarker of inflammation-related carcinogenesis.

### Cancer stem cell markers in inflammation-related carcinogenesis

**Fig. 2:**
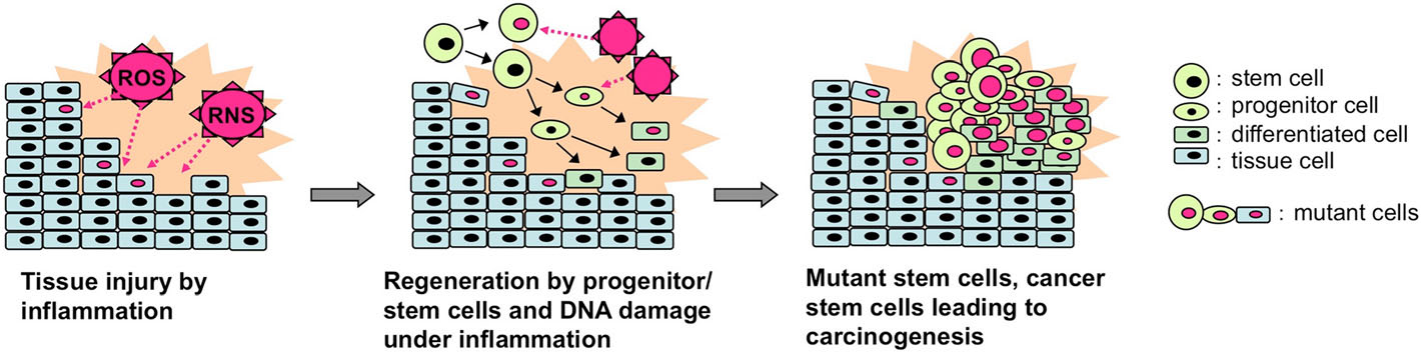
Possible mechanism for generating mutant stem cells by inflammation

The cancer stem cell concept is widely accepted as important for overcoming cancer [23]. Several studies have revealed that cancer stem cells show accumulation of mutations, genetic instability and epigenetic change suggesting that cancer is also a disease of genes. The most important question is how to generate cancer stem cells. Recently, many studies have been reported on the expressions of stemness cell markers in various kinds of cancer. Table 1 summarizes possible markers of cancer stem cells, especially related to each inflammatory causative agent [20]. We reported that 8-nitroguanine was strongly formed at all of these cancer sites from animals and patients with infectious agents. Importantly, we also detected co-localization of 8-nitroguanine and stemness marker in infection-related carcinogenesis, as mentioned in the next section. On the basis of our recent studies, it is considered that chronic inflammation can increase mutagenic DNA lesions through ROS/RNS generation and can promote proliferation via stem cells activation for tissue regeneration (Fig. 2). This idea is also supported by other papers about the association of cancer stem cells with infection and inflammation [24, 25].

### *H. pylori* infection and gastric cancer

**Fig. 3:**
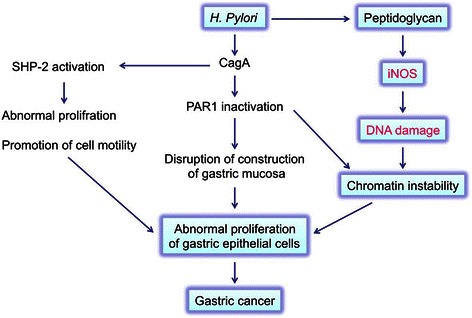
Mechanism of carcinogenesis induced by *H. pylori* infection

The presence of the Gram-negative bacterium, *H. pylori* is associated with not only chronic atrophic gastritis and peptic ulcer but also gastric adenocarcinoma and non-Hodgkin’s lymphoma [mucosa-associated lymphoid tissue (MALT) lymphoma] [26]. *H. pylori* may be responsible for 65–80 % of gastric cancer cases [27]. The mechanisms by which *H. pylori* infection causes gastric cancer have been investigated (Fig. 3). Cytotoxin-associated gene A (CagA) protein is delivered into gastric epithelial cells, and mediates activation of Src homology 2 domain-containing phosphatase 2 (SHP2) tyrosine phosphatase by specifically binding and conformation change, resulting to abnormal proliferation and promotion of cell motility [28]. CagA also play a role in disruption of construction of gastric mucosa by interacting with and inhibiting partitioning-defective 1 (PAR1)/microtubule affinity-regulating kinase (MARK) [29]. Peptidoglycan has been described as a possible factor inducing nucleotide-binding oligomerization domain protein 1 (Nod1)-mediated NF-kB signaling, which can induce iNOS expression [30].

**Fig. 4:**
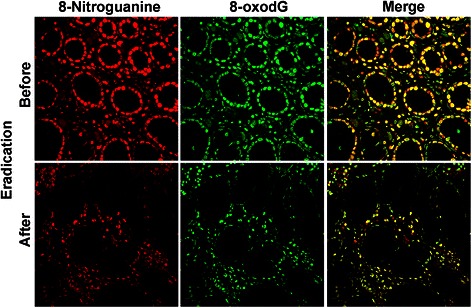
8-Nitroguanine and 8-oxodG formation in gastritis patients with *H. pylori* infection. Double immunofluorescence staining of paraffin sections shows the localization of 8-nitroguanine (red) and 8-oxodG (green) in the gastric epithelium. Yellow colour in right panels (Merge) shows co-localization of 8-nitroguanine and 8-oxodG

We performed a double immunofluorescence labeling study and demonstrated that the intense immunoreactivities of 8-nitroguanine and 8-oxodG were observed both in gastric gland epithelial cells and inflammatory cells in patients with *H. pylori* infection (Fig. 4, upper panels) [31]. Moreover, these immunoreactivities were decreased after eradication (Fig. 4, lower panels). It has been reported that the expression of iNOS was significantly increased in *H. pylori*-positive gastritis compared to *H. pylori*-negative gastritis [32]. These suggest that nitrative and oxidative DNA damage in gastric epithelial cells and their proliferation by *H. pylori* infection may lead to gastric carcinoma.

There are several papers concerning the relation of cancer stem cells in *H. pylori* induced carcinogenesis. *H. pylori* colonize and manipulate the progenitor and stem cell components, which alters turnover kinetics and glandular hyperplasia [33]. *H. pylori* infection and inflammation leads to an epithelial-mesenchymal transition (EMT) and altered tissue regeneration and differentiation from both local epithelial stem cells and bone marrow-derived cells (BMDCs) [34]. These abilities to alter the stem cells may be involved in generating cancer stem cells, in addition to mutagenic DNA damage.

### HBV or HCV infection and liver cancer

HBV or HCV is a major cause of chronic hepatitis, liver cirrhosis, and hepatocellular carcinoma throughout the world [35, 36]. HBV / HCV may account for about 80 % of hepatocellular carcinoma cases [37, 38]. It is generally accepted that hepatocellular carcinoma arises through a multistep process of genetic alterations in hepatocytes during chronic hepatitis C (CHC). However, the mechanism of HCV infection-induced hepatitis followed by hepatocarcinogenesis via DNA damage is still unclear.

**Fig. 5:**
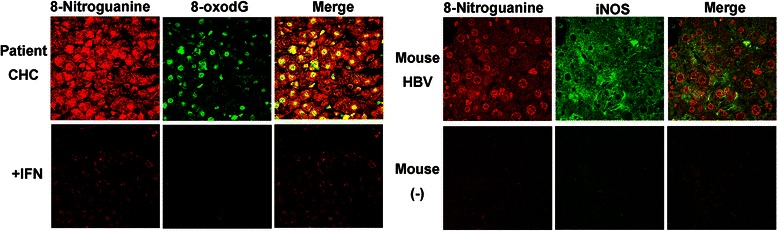
*Left*: 8-Nitroguanine and 8-oxodG accumulation in liver tissues from patients [39]. *Right*: 8-Nitroguanine accumulation and iNOS expression in liver tissues from mice (unpublished data)

We investigated DNA damage in liver biopsy specimens of patients with CHC and the effect of interferon treatment. Immunoreactivities of 8-nitroguanine and 8-oxodG were strongly detected in the liver from patients with CHC in Fig. 5 [39]. 8-Nitroguanine accumulation was found in not only infiltrating inflammatory cells but also hepatocytes particularly in the periportal area. The accumulation of 8-nitroguanine and 8-oxodG increased with inflammatory grade. iNOS expression was observed in the cytoplasm of hepatocytes and Kupffer cells in CHC patients [39]. In the sustained virological responder group after interferon therapy (+INF in Fig. 5a), the accumulation of 8-nitroguanine and 8-oxodG in the liver was markedly decreased [39]. Our results are consistent with the previous reports showing that the expression of iNOS in hepatocytes has been observed in patients with chronic hepatitis [40] and hepatocellular carcinoma [41]. Moreover, we demonstrated the accumulation of 8-nitroguanine and expression of iNOS in liver tissues of mice infected with HBV (Fig. 5). Taken together, these findings indicate that 8-nitroguanine is a useful biomarker to evaluate the severity of HBV/HCV-induced chronic inflammation leading to hepatocellular carcinoma.

It has been reported that hepatic progenitor cells increase in the liver of HCV patients as the disease advances to cirrhosis, while CD133 (stem/progenitor cell marker) -positive cancer stem cells correlated with early recurrence and poor prognosis among HBV related HCC patients [38]. HBV/HCV modulate hypoxic pathways to adapt cells in hypoxic conditions conferring EMT characteristics [38]. Hypoxia sustains the self-renewal characteristics of a portion of cancer cells in hypoxic niches mainly due to the upregulation of Oct4, NANOG, SOX2, Klf4, and c-myc [38]. It is necessary to study whether 8-nitroguanine forms in cancer stem cells.

### Human papillomavirus and cervical cancer

**Fig. 6:**
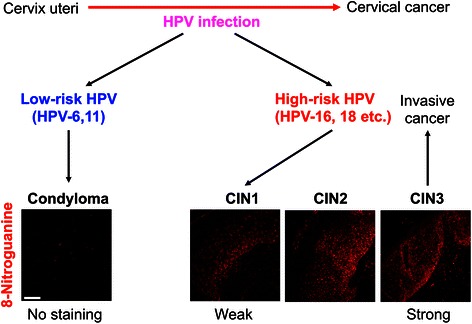
Development of HPV-induced cervical cancer

Cervical cancer is the fourth most common cancer among women worldwide and approximately 70 % of the cases occur in developing countries [42]. HPV may cause about 90 % of cervical cancer cases [43]. Virtually all cases of cervical cancer are attributable to persistent infection with HPV [44]. IARC has evaluated several high-risk types of HPV, including HPV-16, 18, 31, 33, 35, 39, 45, 51, 52, 56, 58 and 59, to be carcinogenic to humans (group 1) [45, 46]. HPV infection is a necessary event that precedes the development of cervical intraepithelial neoplasia (CIN), a premalignant lesion, which partially progresses to cancer [47]. Figure 6 shows a schematic diagram of HPV-induced carcinogenesis. E6 and E7 participate in HPV-induced carcinogenesis by inactivating the tumor suppressor gene products, p53 and Rb, respectively. E6 and E7 expression is necessary but not sufficient to transform the host cell, as genomic instability is required to acquire the malignant phenotype in HPV-initiated cells. Recently, Marullo et al. reported that HPV16 E6 and E7 proteins induced a chronic oxidative stress, to cause genomic instability and increased susceptibility to DNA damage [48]. In addition, inflammation-mediated DNA damage may be involved in cervical carcinogenesis.

Although it is unclear whether HPV infection alone induces the inflammatory responses, epidemiological studies have suggested that cervical inflammation in HPV-infected women is associated with cervical neoplasia [47, 49]. Co-infection with HPV and other pathogens increase the risk of cervical cancer. Among HPV DNA-positive women, infection with herpes simplex virus-2 is associated with the risk of invasive cervical carcinoma. Molecular epidemiological studies have shown that COX-2 is overexpressed in cervical cancer [50, 51]. These findings suggest that inflammation plays a substantial role in HPV-mediated carcinogenesis.

To clarify the role of inflammation-mediated DNA damage in cervical carcinogenesis, we examined 8-nitroguanine formation in cervical biopsy specimens of patients obtained from HPV-infected patients. We compared the extent of 8-nitroguanine formation in patients with different stages of CIN caused by high-risk HPV and condyloma acuminatum, benign cervical warts caused by low-risk HPV. 8-Nitroguanine was formed in the nuclei of atypical epithelial cells of CIN patients but not in condyloma acuminatum patients. Statistical analysis revealed that the staining intensity of 8-nitroguanine was significantly increased in the order of condyloma acuminatum < CIN1 < CIN2-3 [52]. Inflammation-mediated DNA damage, which precedes the genomic abnormalities caused by HPV oncoproteins, may play an important role in carcinogenesis.

**Fig. 7:**
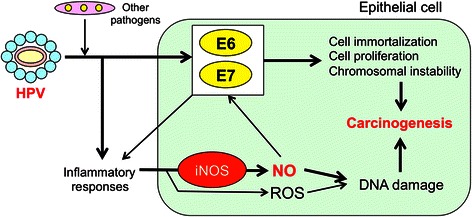
Possible mechanisms of HPV-induced cervical carcinogenesis

The formation of nitrative DNA lesion during cervical carcinogenesis has been supported by a recent study. NO induced DNA damage and increased mutation in HPV-positive human cervical epithelial cell lines established from CIN patients [53]. In addition, NO increased the expression of E6 and E7 genes, resulting in decreased p53 and RB protein levels in these cells [53]. These findings raise the possibility that NO-mediated DNA damage and viral oncoproteins cooperatively contribute to HPV-induced cervical carcinogenesis (Fig. 7).

López et al. reviewed human papillomavirus infections and cancer stem cells of tumors from the uterine cervix [54]. Stem cell associated proteins including human chorionic gonadotropin, the oncogene TP63 and the transcription factor SOX2 were upregulated in samples from women with CIN3 [55]. The stem cell related, cell surface protein podocalyxin was detectable on cells in samples from a subset of women with CIN3. SOX2 and TP63 proteins clearly delineated tumour cells in invasive squamous cervical cancer [55].

### Epstein–Barr virus and nasopharyngeal carcinoma

Lymphomas, gastric cancer and nasopharyngeal carcinoma (NPC) are strongly associated with EBV infection, and account for approximately 1 % of cancer cases worldwide [4]. NPC has a profoundly skewed geographical incidence, being common in the arctic (Inuits and Aleuts), North Africa, and South East Asia [56]. The remarkably higher incidence of NPC among the Chinese, especially in South China and South Eastern Asia is mainly attributed to the non-keratinizing subtype, which has a virtually 100 % association with EBV [4, 56].

**Fig. 8:**
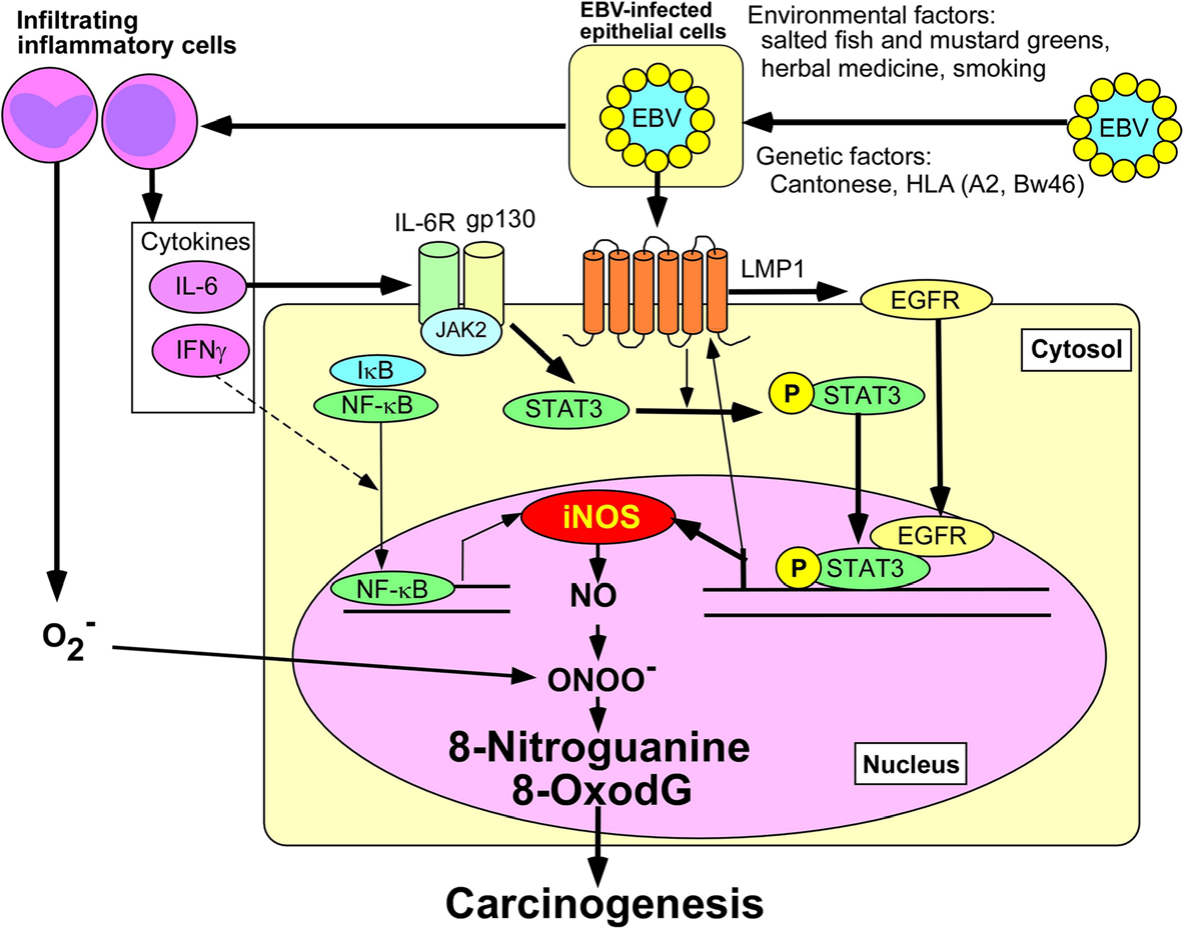
Proposed mechanism of EBV-induced carcinogenesis

We examined 8-nitroguanine and 8-oxodG formation in biopsy specimens from patients with nasopharyngitis and NPC in southern China. 8-Nitroguanine and 8-oxodG were formed in epithelial cells of EBV-positive patients with chronic nasopharyngitis, and their intensities were significantly stronger in cancer cells of NPC patients [57]. The serum level of 8-oxodG in NPC patients was significantly higher than control patients, suggesting the involvement of oxidative stress [58]. We confirmed EBV infection at the nasopharyngeal tissues by using in situ hybridization for EBV-encoded RNAs (EBERs). Also, a viral protein latent membrane protein 1 (LMP1) was detected in cancer cells from all EBV-infected patients. LMP1 induces the expression and nuclear accumulation of epidermal growth factor receptor (EGFR), which in turn interacts with the signal transducer and activator of transcription-3 (STAT3) in the nucleus, leading to transcriptional activation of iNOS [59]. In our study, intensive immunoreactivity of iNOS was detected in the cytoplasm of cancer cells, and EGFR and phosphorylated STAT3 were strongly expressed in cancer cells of NPC patients. Interleukin (IL)-6 was expressed in macrophages of nasopharyngeal tissues of EBV-infected patients. EGFR was accumulated in the nucleus of LMP1-expressing cells, and the addition of IL-6 induced the expression of phosphorylated STAT3 and iNOS and the formation of inflammation-related DNA lesions [3, 57]. The proposed mechanism of EBV-induced carcinogenesis (Fig. 8), that is, EBV infection may induce nuclear accumulation of EGFR and IL-6-mediated STAT3 activation, leading to iNOS expression and formation of 8-nitroguanine and 8-oxodG.

Recently interesting study has been reported [60]. EBV-encoded LMP1 could induce development of CD44-positive stem-like cells in NPC. LMP1 activated and triggered phosphoinositide 3-kinase/protein kinase B (PI3K/AKT) pathway, which subsequently stimulated expression of CD44, development of side population and tumor sphere formation.

### DNA damage and mutant stem cells induced by *Schistosoma haematobium* infection

Chronic infection with SH is associated with urinary bladder cancer, especially in the Middle East and Africa [61]. Contact with contaminated river water is the major risk factor for infection. It is believed that the parasite’s eggs in the host bladder result in irritation, eventual fibrosis and chronic cystitis, leading to carcinogenesis. We demonstrated for the first time that 8-nitroguanine is formed in the tumors of bladder cancer patients with SH infection, by immunohistochemical analysis [21]. The formation of 8-nitroguanine and 8-oxodG was significantly higher in bladder cancer and cystitis tissues than in normal tissues. iNOS expression was co-localized with NF-kB in 8-nitroguanine-positive tumor cells from bladder cancer patients. NF-kB can be activated by TNF-α, a major mediator of inflammation, stimulated by SH egg antigen. These suggest that both 8-nitroguanine and 8-oxodG are formed by iNOS-mediated NO overproduction via NF-kB activation, under SH-caused chronic inflammation.

**Fig. 9:**
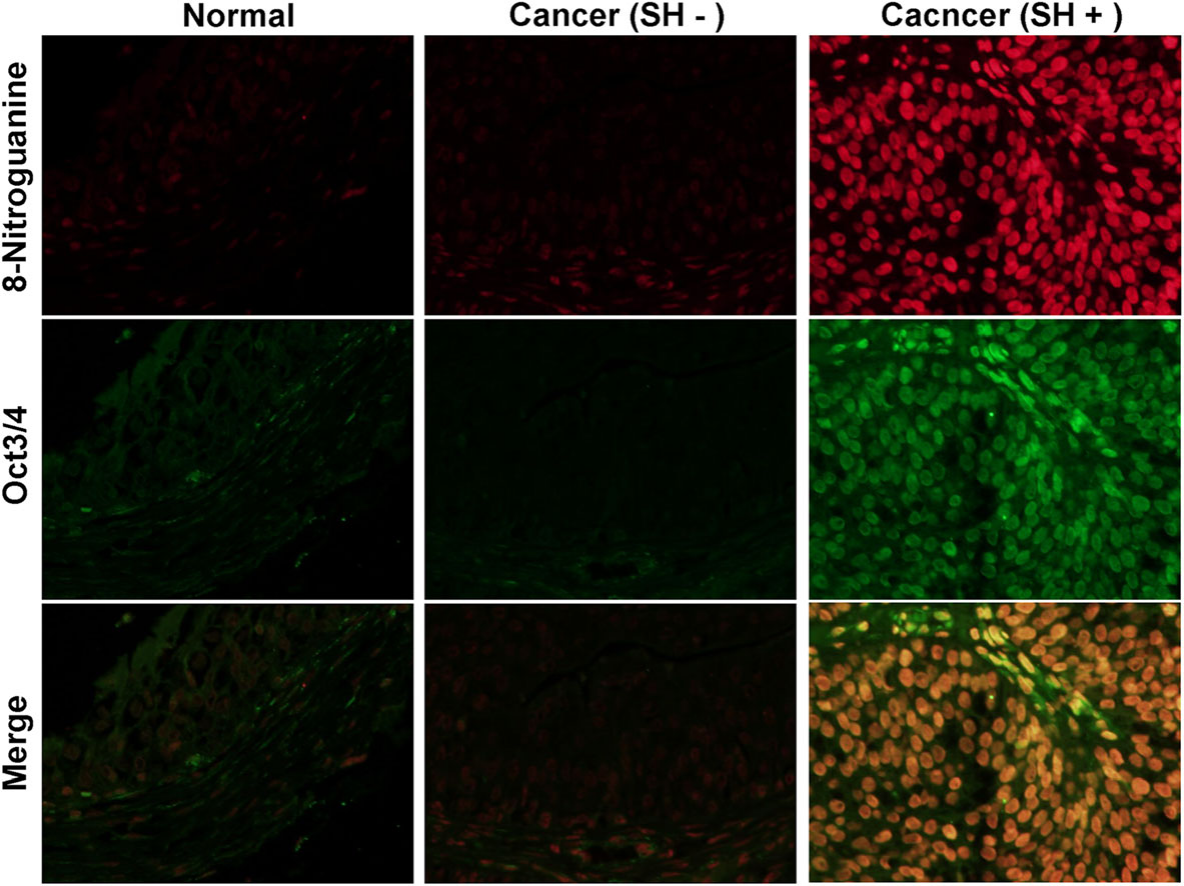
Formation of 8-nitroguanine and expression of Oct3/4 in bladder tissues. The formation of 8-nitroguanine (red) and the expression of Oct3/4 (green) were assessed by double immunofluorescence staining [21]. In the merged image, co-localization of 8-nitroguanine and Oct3/4 is indicated in yellow. Biopsy and surgical specimens were obtained from normal subjects and patients with SH-induced cystitis and bladder cancer. Normal tissues and urinary bladder cancer tissues without SH-infection were obtained from a commercial urinary bladder tissue array (Biomax.us, USA)

A stemness marker, Oct3/4, is necessary for maintaining the self-renewing, cancer stem-like, and chemoradioresistant properties of tumorigenic stem-like cell populations [62, 63], and is thus considered to play roles in the carcinogenesis process. Another stemness marker, CD44, has been identified as a cell surface marker associated with cancer stem cells in tumors [64, 65], including urinary bladder cancer. Expression of CD44v6, a splicing variant of CD44, is correlated with proliferation of poorly differentiated urothelial cells and the characteristic phenotype of tumor-initiating bladder cancer stem cells [66, 67]. Our previous reports have showed that SH-induced urinary bladder cancer correlates with the expression of Oct3/4 [21], while urinary bladder cancer without the infection correlates with the expression of CD44v6 [22]. It is noteworthy that different risk factors induce different levels of expression of stemness markers in urinary bladder carcinoma. Moreover, 8-nitroguanine was formed in Oct3/4-positive stem cells in SH-associated cystitis and cancer tissues as shown in Fig. 9 [21]. Inflammation by SH infection may increase the number of mutant stem cells, in which iNOS-dependent DNA damage occurs via NF-kB activation, leading to tumor development.

### DNA damage and mutant stem cells induced by OV infection

Chronic infection with the liver flukeOV is associated with cholangiocarcinomas (CCA) [5]. Repeated intake of raw fish containing the infective stage of OV is a cause of the parasite-induced CCA. Re-infection with OV is a major risk factor of CCA in northeast Thailand. We assume that OV-associated CCA is one of a model of inflammation-mediated carcinogenesis. We demonstrated 8-nitroguanine and 8-oxodG formation in the bile duct of hamsters fed with metacercariae of OV [68–70]. These DNA lesions were observed in inflammatory cells and epithelium of bile ducts, and their formation increased in a manner dependent on infection times. The anthelminthic drug praziquantel dramatically diminished the DNA lesions and iNOS expression in OV-infected hamsters. Thus, repeated OV-infection can induce the iNOS-dependent nitrative and oxidative damage to nucleic acids in bile ducts via NF-$k$B expression, which may participate in CCA.

In our study with patients, the formation of 8-oxodG and 8-nitroguanine occurred to a much greater extent in cancerous tissues than in non-cancerous tissues in CCA patients, indicating that these DNA lesions contribute to tumor initiation [71]. Urinary 8-oxodG levels were significantly higher in CCA patients than in OV–infected patients, and higher in OV–infected subjects than in healthy subjects. The urinary 8-oxodG levels in OV–infected patients significantly decreased two months after praziquantel treatment [72].

Our study with proteomics approach showed that oxidation of serotransferrin, alpha-1-antitrypsin (A1AT) and heat shock protein 70-kDa protein 1 (HSP70.1) were significantly associated with poor prognoses [73]. HSP70.1 acts as a molecular chaperone to protect various cells from oxidative stress. A1AT, a glycoprotein, is a member of the serpins (serine protease inhibitors), inhibitors of a wide variety of proteases in relation to tumor invasion. Serotransferrin (transferrin) is an iron (Fe^3+^)-binding and -transporting protein. Interestingly, we observed that serotransferrin was highly expressed and co-localized with iron in the tumor, suggesting iron accumulation and its release from oxidatively-damaged serotransferrin. We have proposed that oxidative damage of serotransferrin, HSP70.1 and A1AT may induce oxidative stress by iron-accumulation and dysfunction of anti-oxidative and anti-invasive properties, leading to increased oxidative DNA damage and progression of CCA.

**Fig. 10:**
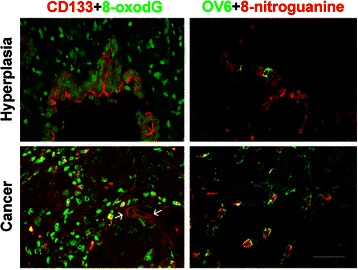
Colocalization of stem cell markers and DNA damage. Double-immunofluorescence staining of stem/progenitor cell markers (CD133 and OV6) and DNA lesions (8-oxodG and 8-nitroguanine) in cholangiocarcinoma tissues. White arrows indicate co-localization of DNA damage marker and stemness marker in cancer cells. Original magnification is × 400; Scale bar = 25 μm

Recently, we observed high expression and co-localization of hepatocyte marker and cholangiocyte marker in OV-associated CCA patients, suggesting the involvement of stem cells in CCA development [74]. Stem/progenitor cell markers (CD133 and OV6) were positively stained in CCA cases (Fig. 10). Quantitative analysis of 8-oxodG revealed significantly increased levels in CD133- and/or Oct3/4-positive tumor tissues compared to negative tumor tissues, suggest that CD133 and Oct3/4 in CCA are associated with increased formation of DNA lesions [74]. Moreover, CD133- and Oct3/4-positive CCA patients had significant associations with poor prognoses. These findings suggest that CD133 and Oct3/4 in CCA are highly associated with formation of DNA lesions, which may be involved in mutant stem cells, leading to cancer stem cells. Inflammation by OV infection may increase the number of mutant stem cell under oxidative and nitrative stresses, and the mutant stem cell proliferation may promote to be cancer stem cells of CCA.

## Conclusions

Nitrative and oxidative DNA lesions with mutagenic properties are formed in various types of inflammation-related cancer tissues. We have proposed a mechanism for the generation of cancer stem cells by inflammation in Fig. 2. Chronic inflammation by infectious agents, inflammatory diseases, and other factors causes various types of damage to nucleic acids, proteins, tissue and so on, via ROS/RNS generation. Tissue injury under chronic inflammation may activate progenitor/stem cells for regeneration. In these cells, ROS/RNS from inflammation can cause multiple mutations, which may generate mutant stem cells and cancer stem cells, leading to carcinogenesis. Indeed, 8-nitroguanine was formed in stemness marker-positive cells in parasite-associated cancer tissues. The mechanism for generation of cancer stem cells will be explained by our ongoing studies on the formation of 8-nitroguanine in stem-like cells of target tissues associated with other inflammation-related cancers.

## Abbreviations

8-oxodG: 8-oxo-7,8-dihydro-2’-deoxyguanosine
A1AT: Alpha-1-antitrypsin
BMDCs: Bone marrow-derived cells
CagA: Cytotoxin-associated gene A
CCA: Cholangiocarcinomas
CHC: Chronic hepatitis C
CIN: Cervical intraepithelial neoplasia
CS: *Clonorchis sinensis*
EBERs: EBV-encoded RNAs
EBV: Epstein-Barr virus
EGFR: Epidermal growth factor receptor
EMT: Epithelial-mesenchymal transition
eNOS: Endothelial NO synthase
*H. pylori*: *Helicobacter pylori*
HBV: Hepatitis B virus
HCV: Hepatitis C virus
HIV-1: Human immunodeficiency virus-1
HPV: Human papillomavirus
HSP70.1: Heat shock protein 70-kDa protein 1
HTLV-1: Human T-cell lymphotropic virus type 1
IARC: International Agency for Research on Cancer
IL: Interleukin
INF: Interferon therapy
iNOS: Inducible NO synthase
LMP1: Latent membrane protein 1
MALT: Mucosa-associated lymphoid tissue
MARK: Microtubule affinity-regulating kinase
nNOS: Neuronal NO synthase
NO: Nitric oxide
Nod1: Nucleotide-binding oligomerization domain protein 1
NPC: Nasopharyngeal carcinoma
O_2_^−^: Superoxide
ONOO^−^: Peroxynitrite
OV: *Opisthorchis viverrini*
PAR1: Partitioning-defective 1
PI3K/AKT: Phosphoinositide 3-kinase/protein kinase B
RNS: Reactive nitrogen species
ROS: Reactive oxygen species
SH: *Schistosoma haematobium*
SHP2: Src homology 2 domain-containing phosphatase 2
STAT3: Signal transducer and activator of transcription-3
TNF-α: Tumor necrosis factor-α
